# The Tumor Suppressor p53 in Mucosal Melanoma of the Head and Neck

**DOI:** 10.3390/genes8120384

**Published:** 2017-12-13

**Authors:** Marie Kristin Fritsche, Andreas Knopf

**Affiliations:** Department of Otorhinolaryngology/Head and Neck Surgery, Technische Universität München, Ismaningerstr, 22, 81675 München, Germany; a.knopf@lrz.tu-muenchen.de

**Keywords:** mucosal melanoma, p53, Puma, N-ras, pathway, apoptosis

## Abstract

Despite worldwide prevention programs, the incidence for cutaneous melanoma is continuously increasing. Mucosal melanoma (MM) represents a rare but highly aggressive phenotype of common melanoma with predilection in the sinonasal system. Far away from ultraviolet sun exposure, the molecular mechanisms underlying tumorigenesis and the highly aggressive clinical behavior are poorly understood. In many solid malignomas of the head and neck region, p53 tumor suppressor functions as oncogene due to p53 protein stabilizing mutation. Interestingly, the vast majority of MM demonstrates constitutively expressed p53 protein, with protein stabilizing mutations being rare. Abrogated activation of p53 target genes results in derogation of the apoptotic signal cascade and contributes to the strong resistance against chemotherapeutic agents activating p53 dependent apoptosis. The current review illustrates the role of p53 and its pathway in MM.

## 1. Introduction

Mucosal melanoma is a rare but highly aggressive phenotype of melanomas representing 1–5% of the overall cohort [1,2,3]. Mucosal melanomas (MMs) originate from melanocytes of the mucosal surfaces in the eye, epithelia of sinonasal system, oropharynx and anus. The majority of MM is located in the head and neck region and most commonly found in the nasal cavity, paranasal sinuses and oral cavity [4,5,6]. Melanocytes which function as ultraviolet (UV)-light protector in the skin are peculiarly found in the mucosa. In the oral mucosa, melanocytes can be found from 20 weeks of gestation [7]. Their function is not fully understood, but it seems that these melanocytes have antimicrobial and immunological functions, such as antigen presentation and cytokine production [8]. MM demonstrates a dramatically decreased 5-year survival rate (17%) when compared with their cutaneous (CM) counterparts in early disease stages (80%) [2]. The Union for National Cancer Control (UICC) staging system takes the poor prognosis into account and relinquishes T1/2 status. Poor prognosis in MM might be explained by the close localization to the orbit, skull base and brain, leading to a difficult operability with insufficient R-status [9,10]. In contrast, lymph node positivity and distant metastasis that usually result in advanced UICC disease stages occur infrequently. At time of diagnosis, only 10–20% of MM patients exhibit lymph node metastases and also tend to exhibit distant metastases less frequently [9,10]. Molecular mechanisms in MM that contribute to the highly aggressive phenotype remain unclear [11]. 

## 2. Physiology of the p53 Tumor Suppressor 

**Figure 1:**
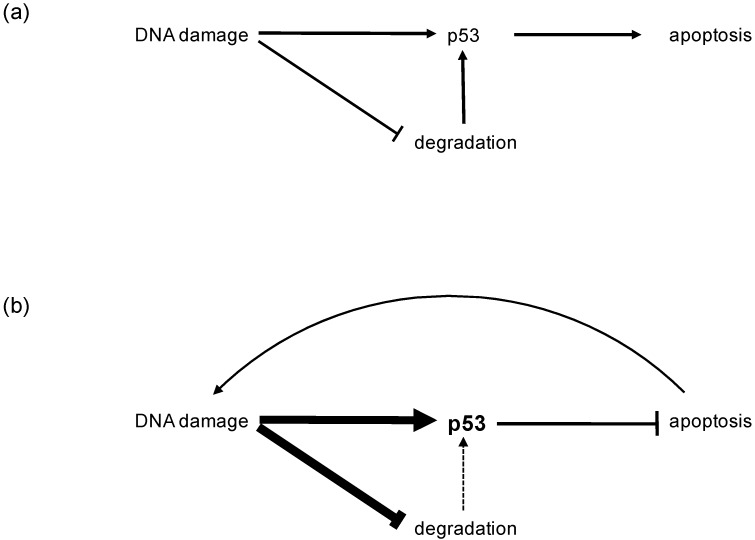
Normal and feedback regulation of p53: (**a**) Usually, after cellular stresses p53 stabilization is regulated through posttranslational modifications (PTMs) and inhibition of proteasomal degradation, resulting in apoptosis. (**b**) Derogation of the p53 depended apoptotic cascade results in activation of positive feedback loops that enhance (fat arrows) p53 stabilization and inhibition of degradation to induce apoptosis. Thus, accumulation of wildtype p53 and nonregulation of apoptosis results from lack of function.

The p53 tumor suppressor gene (*TP53*) is the most common mutated gene in solid cancers harboring mutated p53 (mtp53) in 42–50% of tumor specimens [12,13]. The p53’s function as a transcription factor is crucial for deciding cell fate, by activating growth arrest, cellular senescence, DNA repair or apoptotic signal pathways. After DNA damage and oxidative stress p53 is activated through blockage of Mdm2 (mouse double minute 2), which is responsible for p53 degradation [14]. Further posttranslational modifications (PTM) such as acetylation, methylation and phosphorylation will lead to enhanced p53 protein stability and increased site–specific DNA binding. The p53 binds as tetrameric complex to p53 responsive elements and recruits cofactors to mediate transcriptional induction or repression [15]. Under physiological conditions, latent expression of p53 has no effect on cell survival, cell cycle regulation, or transcription rate. Accumulation of active p53 is predominantly mediated by PTM in response to cellular stresses [16]. Among these PTMs, the blockage of Mdm2 is achieved through Thr18 phosphorylation. Additionally, p53 stabilization is induced by phosphorylation of Ser15 and Ser20 through stress-induced kinases ataxia telangiectasia mutated (ATM), ATR, checkpoint kinase 1 (Chk1), Chk2 and DNA-dependent protein kinase (DNA-PK), probably leading to inhibition of Mdm2 binding [14]. Interestingly, several feedback loops regulate the p53 pathway, including p53 dependent proteins who themselves are influencing the activation (phosphatase and tensin homolog (PTEN)-Akt , p14/19 alternative reading frame (ARF) and retinoblastoma (Rb)) or the downregulation (Mdm2, Cop-1, Pirh-2, p73deltaN, Cyclin G, wild-type p53 unduced phosphatase 1 (wip-1) and Siah-1 (seven in absentia homolog 1)) of p53 [17]. Therefore, derogation of p53 apoptosis, growth arrest or DNA repair downstream pathways will subsequently result in the activation of positive feedback loops and stabilization of wildtype p53 (Figure 1) [18]. Knowledge about the interconnections between signal transduction pathways will be of major importance to understand the role of the p53 tumor suppressor gene in MM.

## 3. Genetic Aberrations in Mucosal Melanoma

In comparison to squamous cell carcinomas, the most frequent histology in mucosal head and neck malignancy, both CM and MM show distinct mutational landscape. The mutational load in MM was significantly lower when compared with their cutaneous counterparts [12]. In a cohort of CM and MM investigated by Ragnarsson-Olding et al. p53 mutations occurred in 18% of CM and in 29% of MM (half of them were silent). There were no differences in CC/TT tandem mutations, that were considered to be typical UV-induced lesions. N-ras mutations were detected in 32% of CM derived from sun-exposed head and neck areas, but only in 7% of MM, suggesting UV-radiation induces N-ras but not TP53 mutations [19]. Interestingly, Gwosdz et al. detected an accumulation of the p53 protein in most of the CM and MM of the head and neck region (71% and 58%), whereas mutations that lead to protein stabilization were found in 14% of CM specimens but not in MM [20].

## 4. p53 Protein Expression in Mucosal Melanoma

**Figure 2:**
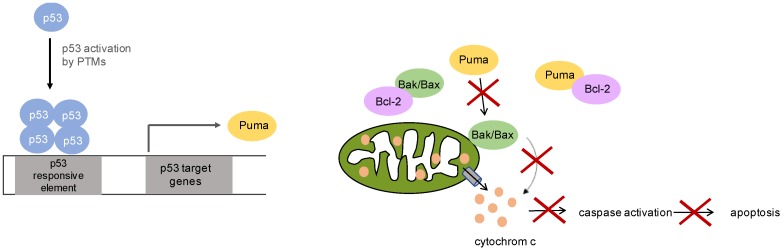
Function of p53 upregulated modulator of apoptosis (Puma) in apoptosis: After activation of p53, its target gene Puma is transcribed. Puma functions in mediating apoptosis through indirect activation of Bcl2 associated X/Bcl2 antagonist/Killer (Bax/Bak) through inhibiting Bcl-2 family member proteins or through direct activation of Bax/Bak, which mediate mitochondrial outer membrane permeabilization and thereby releasing cyctochrome c and activating apoptosis. Puma plays a critical role in mediating p53 mediated induction of apoptosis and non-functional Puma will lead to an obstructed apoptosis.

Regarding the findings that wildtype p53 is rapidly degraded and solely stabilized through posttranslational modifications after stress induction, one can assume that immunhistochemical (IHC) detection of p53 is based on its mutational prolonged stability. Accumulation of wildtype p53 protein ranged from 50–94% in CM and 21–80% in MM [10,19,20,21,22]. Different p53 expression levels refer, most likely, to different cut-off levels in the underlying cohort ranging from 1–25% of immunohistochemically stained tumor cells [19]. All studies failed to achieve statistical significance between different staining pattern in MM and CM. However, several studies could not identify a concordance between p53 mutations and p53 protein expression in MM or in CM. Accordingly, the accumulation of p53 detected in IHC despite the low mutation rate can be explained by a loss of p53 function and the resulting activated positive feedback loop (Figure 2). This stabilizes p53 through inhibition of degradation and protein stabilization by PTMs. Therefore, it is not surprising that p53 positive tumors showed reduced overall and disease specific survival [22,23,24] highlighting the impact of a derogated p53 dependent apoptosis in the tumorigenesis of both MM and CM.

## 5. p53 Signaling in Mucosal Melanoma

Although wildtype p53 is present in most CM and MM, chemotherapeutics targeting the p53 pathway such as cisplatin or carboplatin are barely successful suggesting abrogated functional integrity of the accumulated protein [25]. The principle cellular antagonist of p53 is Mdm2, an E3 ubiquitin ligase which recruits p53 to nuclear and cytoplasmic proteasomes for degradation by mono-ubiquitinylation [26]. In malignant melanoma, neither mutations of Mdm2, nor dysfunctional Mdm2-p53 interaction have been found. Moreover, the signaling pathway from DNA damage to activation of p53 by using cisplatin as pathway inductor is unaffected [27]. Considering the p53 downstream pathway in malignant melanoma cells compared to melanocytes, a notably lower expression of cyclin dependent kinase inhibitor 1A (*CDKN1A*), growth arrest and DNA damage inducible alpha (*GADD45A*) and *BBC3* (Bcl2 binding component 3, also p53 upregulated modulator of apoptosis) mRNA expression was observed, corroborating the hypothesis of a disrupted p53 dependent apoptosis [27]. In previous experiments we showed, that p53 target genes *BBC3*, Bcl2 associated X (*BAX*) and caspase 9 (*CASP9*) had no distinctions in mRNA and protein expression in formalin-fixed paraffin-empedded tissue (FFPE) samples and untreated melanoma cells of cutaneous and mucosal origin (except for a MM with a higher BAX mRNA expression). Notably, there were no differences in *CDK1*/Cdk1 (cyclin dependent kinase 1) expression detectable in FFPE samples and cells in IHC, Western Blot and qRT-PCR. However, after cisplatin treatment, only MM cells decreased Cdk1 expression, whereas CM stabilized the Cdk1 expression and thereby maintaining cell cycle progression. But most interestingly was the observation that p53 upregulated modulator of apoptosis (Puma) failed to be detected in MM cells by Western Blot experiments, whereas Puma protein was detectable in FFPE samples in some patients. However, in this study a positive staining was defined as greater than 10% positive cells [10]. They did not quantify the staining intensity, as loss of Puma expression may be a marker for malignant transformation [28]. This protein is a member of the BH3-only Bcl-2 family, and is one of the most potent inductor of apoptosis among this protein family. Puma is expressed at low levels under normal conditions and can be rapidly induced transcriptionally by p53. p53 is recruited to the two p53 responsive elements in the Puma promotor, in which both p53 and its binding sites on the promoter are indispensable. The mechanism of Puma mediated apoptosis includes the indirect activation of the pro-apoptotic proteins Bax and/or Bak through interaction with the anti-apoptotic Bcl-2 family members, and/or the direct activation of Bax/Bak. Nevertheless, both mechanisms lead to mitochondrial dysfunction and caspase activation, ultimately leading to apoptosis (Figure 2) [29]. It was not only shown that Puma expression is significantly reduced in melanoma and inversely correlated with disease progression, but is also shown to be less expressed in MM. Therefore, one can assume Puma as a marker for disease aggressiveness [10,28]. There is different research done in other tumor entities which could explain the reduced Puma expression and therefore the lacking ability to induce apoptosis. It was shown that Puma is a target of autophagy in CM and that apoptosis can be induced through restoration of Puma expression through Chloroquine [30,31,32]. Furthermore, it was reported that the miRNAs miR-221 and miR-222 target Puma to maintain cell proliferation in glioblastoma and epithelial cancers, [33,34]. So far, in MM Puma seems to play a significant role in apoptosis induction and the regulation of its expression needs to be investigated further. Nevertheless, more research needs to be done so that Puma can be targeted in therapy to overcome resistance to chemotherapy and improve the patient’s prognosis.

## 6. Conclusions

In conclusion, due to the low mutation load in MM and the poor response against chemotherapy, the low expression and missing inducibility of Puma seems to be of further interest. Despite lacking apoptosis, the transcriptional function of p53 seems to be functionally intact, as it was approved by induction of mRNA expression of its targets. This leads to the hypothesis, that a missing Puma functionality might be responsible for the poor response to chemotherapy. This hypothesis is supported by the fact, that reduced Puma expression was observed in melanoma and that the Puma expression inversely correlates with disease progression. Therefore, targeting Puma in therapy might overcome resistance to chemotherapy and improve the patient’s prognosis.

## References

[1] Kim H.S., Jung M., Kang H.N., Kim H., Park C.W., Kim S.M., Shin S.J., Kim S.H., Kim S.G., Kim E.K. (2017). Oncogenic BRAF fusions in mucosal melanomas activate the MAPK pathway and are sensitive to MEK/PI3K inhibition or MEK/CDK4/6 inhibition. Oncogene.

[2] Lengyel E., Gilde K., Remenár E., Esik O. (2003). Malignant mucosal melanoma of the head and neck. Pathol. Oncol. Res..

[3] Papaspyrou G., Garbe C., Schadendorf D., Werner J.A., Hauschild A., Egberts F. (2011). Mucosal melanomas of the head and neck: New aspects of the clinical outcome, molecular pathology, and treatment with c-kit inhibitors. Melanoma Res..

[4] Mendenhall W.M., Amdur R.J., Hinerman R.W., Werning J.W., Villaret D.B., Mendenhall N.P. (2005). Head and neck mucosal melanoma. Am. J. Clin. Oncol..

[5] Tas F., Keskin S., Karadeniz A., Dagoglu N., Sen F., Kilic L., Yildiz I. (2011). Noncutaneous melanoma have distinct features from each other and cutaneous melanoma. Oncology.

[6] Frakes J.M., Strom T.J., Naghavi A.O., Trotti A., Rao N.G., McCaffrey J.C., Otto K.J., Padhya T., Caudell J.J. (2016). Outcomes of mucosal melanoma of the head and neck. J. Med. Imaging Radiat. Oncol..

[7] Lourenco S.V., Fernandes J.D., Hsieh R., Coutinho-Camillo C.M., Bologna S., Sangueza M., Nico M.M. (2014). Head and neck mucosal melanoma: A review. Am. J. Dermatopathol..

[8] Mackintosh J.A. (2001). The antimicrobial properties of melanocytes, melanosomes and melanin and the evolution of black skin. J. Theor. Biol..

[9] Thierauf J., Veit J., Doscher J., Theodoraki M.N., Greve J., Hoffmann T.K. (2015). Mucosal melanoma of the head and neck. Laryngorhinootologie.

[10] Fritsche M.K., Metzler V., Becker K., Plettenberg C., Heiser C., Hofauer B., Knopf A. (2015). Cisplatin fails to induce puma mediated apoptosis in mucosal melanomas. Oncotarget.

[11] Mihajlovic M., Vlajkovic S., Jovanovic P., Stefanovic V. (2012). Primary mucosal melanomas: A comprehensive review. Int. J. Clin. Exp. Pathol..

[12] Kandoth C., McLellan M.D., Vandin F., Ye K., Niu B., Lu C., Xie M., Zhang Q., McMichael J.F., Wyczalkowski M.A. (2013). Mutational landscape and significance across 12 major cancer types. Nature.

[13] Soussi T., Ishioka C., Claustres M., Beroud C. (2006). Locus-specific mutation databases: Pitfalls and good practice based on the p53 experience. Nat. Rev. Cancer.

[14] Brooks C.L., Gu W. (2010). New insights into p53 activation. Cell Res..

[15] Riley T., Sontag E., Chen P., Levine A. (2008). Transcriptional control of human p53-regulated genes. Nat. Rev. Mol. Cell Biol..

[16] Oren M. (1999). Regulation of the p53 tumor suppressor protein. J. Biol. Chem..

[17] Harris S.L., Levine A.J. (2005). The p53 pathway: Positive and negative feedback loops. Oncogene.

[18] Blagosklonny M.V. (1997). Loss of function and p53 protein stabilization. Oncogene.

[19] Ragnarsson-Olding B.K., Karsberg S., Platz A., Ringborg U.K. (2002). Mutations in the TP53 gene in human malignant melanomas derived from sun-exposed skin and unexposed mucosal membranes. Melanoma Res..

[20] Gwosdz C., Scheckenbach K., Lieven O., Reifenberger J., Knopf A., Bier H., Balz V. (2006). Comprehensive analysis of the p53 status in mucosal and cutaneous melanomas. Int. J. Cancer.

[21] Ragnarsson-Olding B., Platz A., Olding L., Ringborg U. (2004). p53 protein expression and TP53 mutations in malignant melanomas of sun-sheltered mucosal membranes versus chronically sun-exposed skin. Melanoma Res..

[22] Prasad M.L., Patel S.G., Shah J.P., Hoshaw-Woodard S., Busam K.J. (2012). Prognostic significance of regulators of cell cycle and apoptosis, p16^INK4a^, p53, and bcl-2 in primary mucosal melanomas of the head and neck. Head Neck Pathol..

[23] Prasad M.L., Patel S.G., Huvos A.G., Shah J.P., Busam K.J. (2004). Primary mucosal melanoma of the head and neck: A proposal for microstaging localized, Stage I (lymph node-negative) tumors. Cancer.

[24] Chen H., Li Y., Long Y., Tang E., Wang R., Huang K., Xie C., Chen G. (2017). Increased p16 and p53 protein expression predicts poor prognosis in mucosal melanoma. Oncotarget.

[25] Bhatia S., Tykodi S.S., Thompson J.A. (2009). Treatment of metastatic melanoma: An overview. Oncology.

[26] Moll U.M., Petrenko O. (2003). The MDM2-p53 interaction. Mol. Cancer Res..

[27] Knopf A., Plettenberg C., Pickhard A., Bas M., Reifenberger J., Bier H., Balz V. (2011). Analysis of the functional integrity of the p53 tumor-suppressor gene in malignant melanoma. Melanoma Res..

[28] Karst A.M., Dai D.L., Martinka M., Li G. (2005). PUMA expression is significantly reduced in human cutaneous melanomas. Oncogene.

[29] Yu J., Zhang L. (2008). PUMA: A potent killer with or without p53. Oncogene.

[30] Amaravadi R.K. (2013). PUMA: A puzzle piece in chloroquine's antimelanoma activity. J. Investig. Dermatol..

[31] Lakhter A.J., Sahu R.P., Sun Y., Kaufmann W.K., Androphy E.J., Travers J.B., Naidu S.R. (2013). Chloroquine promotes apoptosis in melanoma cells by inhibiting BH3 domain-mediated PUMA degradation. J. Investig. Dermatol..

[32] Egger M.E., Huang J.S., Yin W., McMasters K.M., McNally L.R. (2013). Inhibition of autophagy with chloroquine is effective in melanoma. J. Surg. Res..

[33] Zhang C.Z., Zhang J.X., Zhang A.L., Shi Z.D., Han L., Jia Z.F., Yang W.D., Wang G.X., Jiang T., You Y.P. (2010). MiR-221 and miR-222 target PUMA to induce cell survival in glioblastoma. Mol. Cancer.

[34] Zhang C., Zhang J., Zhang A., Wang Y., Han L., You Y., Pu P., Kang C. (2010). PUMA is a novel target of miR-221/222 in human epithelial cancers. Int. J. Oncol..

